# Cyclin D1 represses peroxisome proliferator-activated receptor alpha and inhibits fatty acid oxidation

**DOI:** 10.18632/oncotarget.10274

**Published:** 2016-06-24

**Authors:** Sushama Kamarajugadda, Jennifer R. Becker, Eric A. Hanse, Douglas G. Mashek, Mara T. Mashek, Anna M. Hendrickson, Lisa K. Mullany, Jeffrey H. Albrecht

**Affiliations:** ^1^ Gastroenterology Division, Minneapolis VA Health Care System, Minneapolis, MN 55417, USA; ^2^ Minneapolis Medical Research Foundation, Minneapolis, MN, 55404, USA; ^3^ Department of Biochemistry, Molecular Biology, and Biophysics, University of Minnesota, Minneapolis, MN 55455, USA

**Keywords:** breast cancer, hepatocellular carcinoma, liver regeneration, metabolism, peroxisome proliferator-activated receptor alpha

## Abstract

Cyclin D1 is a cell cycle protein that promotes proliferation by mediating progression through key checkpoints in G1 phase. It is also a proto-oncogene that is commonly overexpressed in human cancers. In addition to its canonical role in controlling cell cycle progression, cyclin D1 affects other aspects of cell physiology, in part through transcriptional regulation. In this study, we find that cyclin D1 inhibits the activity of a key metabolic transcription factor, peroxisome proliferator-activated receptor α (PPARα), a member of nuclear receptor family that induces fatty acid oxidation and may play an anti-neoplastic role. In primary hepatocytes, cyclin D1 inhibits PPARα transcriptional activity and target gene expression in a cdk4-independent manner. In liver and breast cancer cells, knockdown of cyclin D1 leads to increased PPARα transcriptional activity, expression of PPARα target genes, and fatty acid oxidation. Similarly, cyclin D1 depletion enhances binding of PPARα to target sequences by chromatin immunoprecipitation. In proliferating hepatocytes and regenerating liver *in vivo*, induction of endogenous cyclin D1 is associated with diminished PPARα activity. Cyclin D1 expression is both necessary and sufficient for growth factor-mediated repression of fatty acid oxidation in proliferating hepatocytes. These studies indicate that in addition to playing a pivotal role in cell cycle progression, cyclin D1 represses PPARα activity and inhibits fatty acid oxidation. Our findings establish a new link between cyclin D1 and metabolism in both tumor cells and physiologic hepatocyte proliferation.

## INTRODUCTION

During the processes of proliferation and growth, cells undergo marked shifts in metabolism in order to provide for the synthesis of new cellular components, energy production, and maintenance of cellular redox balance. These changes typically include increased utilization of glucose and glutamine and decreased oxidative phosphorylation. Similar alterations are seen in malignant cells, and the connection between cancer and metabolism is an area of growing interest [1–5]. However, the full spectrum and regulation of metabolic changes seen in proliferation and malignancy are incompletely understood.

The liver provides a unique *in vivo* setting to study the interplay between cell metabolism, growth, and proliferation. A primary role of the liver is to regulate systemic energy homeostasis through the synthesis and catabolism of lipids, glucose, proteins, and other substrates. Hepatocytes rarely replicate under normal conditions, but these cells readily proliferate in response to acute and chronic liver injuries. During the process of compensatory hepatocyte proliferation and growth, the metabolic function of the liver is altered to accommodate the synthetic and energetic demands of these processes [6, 7]. The mechanisms underlying the adaptations in hepatic metabolism during liver regeneration are poorly understood, but include changes in the systemic hormonal milieu and regulation of key metabolic transcription factors including the nuclear receptors hepatocyte nuclear factor 4α (HNF4α) and NR1H4 (FXR).

The cell cycle is controlled by protein complexes consisting of cyclins and cyclin-dependent kinases (cdks) [4]. In many types of cells (including hepatocytes), cyclin D1 is induced during G1 phase by extracellular mitogenic signals and activates cdk4 (or cdk6). The best-defined role of cyclin D1/cdk4 is to phosphorylate the retinoblastoma (Rb) and related proteins, leading to progression through the late G1 restriction point(s) and activation of cyclin/cdk complexes acting downstream in the cell cycle. Expression of cyclin D1 alone is sufficient to promote hepatocyte proliferation and liver growth, even under conditions that are normally inhibitory [8–12].

In addition to its role in normal proliferation, cyclin D1 is one of the most frequently over-expressed oncogenes in human cancers [13], and abundant literature has demonstrated that it can play an important role in cancer development. Cyclin D1 also regulates cell physiology independently of cdks, and emerging evidence suggests that it can play a major role in transcriptional control [14, 15]. For example, cyclin D1 has been shown to bind and regulate the activity of several members of the nuclear receptor transcription factor family including androgen receptor, estrogen receptor α, HNF4α, peroxisome proliferator-activated receptor (PPAR) γ, and thyroid hormone receptor β [16–20]. Cyclin D1 also regulates transcriptional co-regulators including p300 and C-terminal Binding Protein (CBP) [14, 21, 22]. Thus, in addition to its canonical role in promoting cell cycle progression, cyclin D1 can regulate cell metabolism via its interaction with transcription factors [23].

In this study, we demonstrate that cyclin D1 inhibits the activity of PPARα, a nuclear receptor that plays a major role in lipid catabolism and energy utilization [24]. Cyclin D1 down-regulates the expression of numerous PPARα target genes and inhibits fatty acid (FA) oxidation in both hepatic cells and in a variety of cancer cell lines. Thus, in addition to being a pivotal mediator of cell cycle progression, cyclin D1 acts as a metabolic switch in proliferating and malignant cells.

## RESULTS

Cyclin D1 is not normally expressed in the liver of adult male mice, but this protein is markedly induced by injuries that promote hepatocyte proliferation (such as 70% partial hepatectomy) and by mitogens in primary hepatocytes [6, 8, 10]. Transient transduction of cyclin D1 into hepatocytes *in vivo* is sufficient to promote robust hepatocyte proliferation and liver growth in the absence of other stimuli. In a prior study, we performed gene array analysis of livers following cyclin D1 transduction [25], and found that this protein regulated several pathways involved in metabolism. On further analysis of this data (Supplementary Figure S1), we noted that cyclin D1 inhibited the expression of numerous of PPARα target genes (with a Z-score of −2.466, *p* = 0.016), leading to the current study.

**Figure 1:**
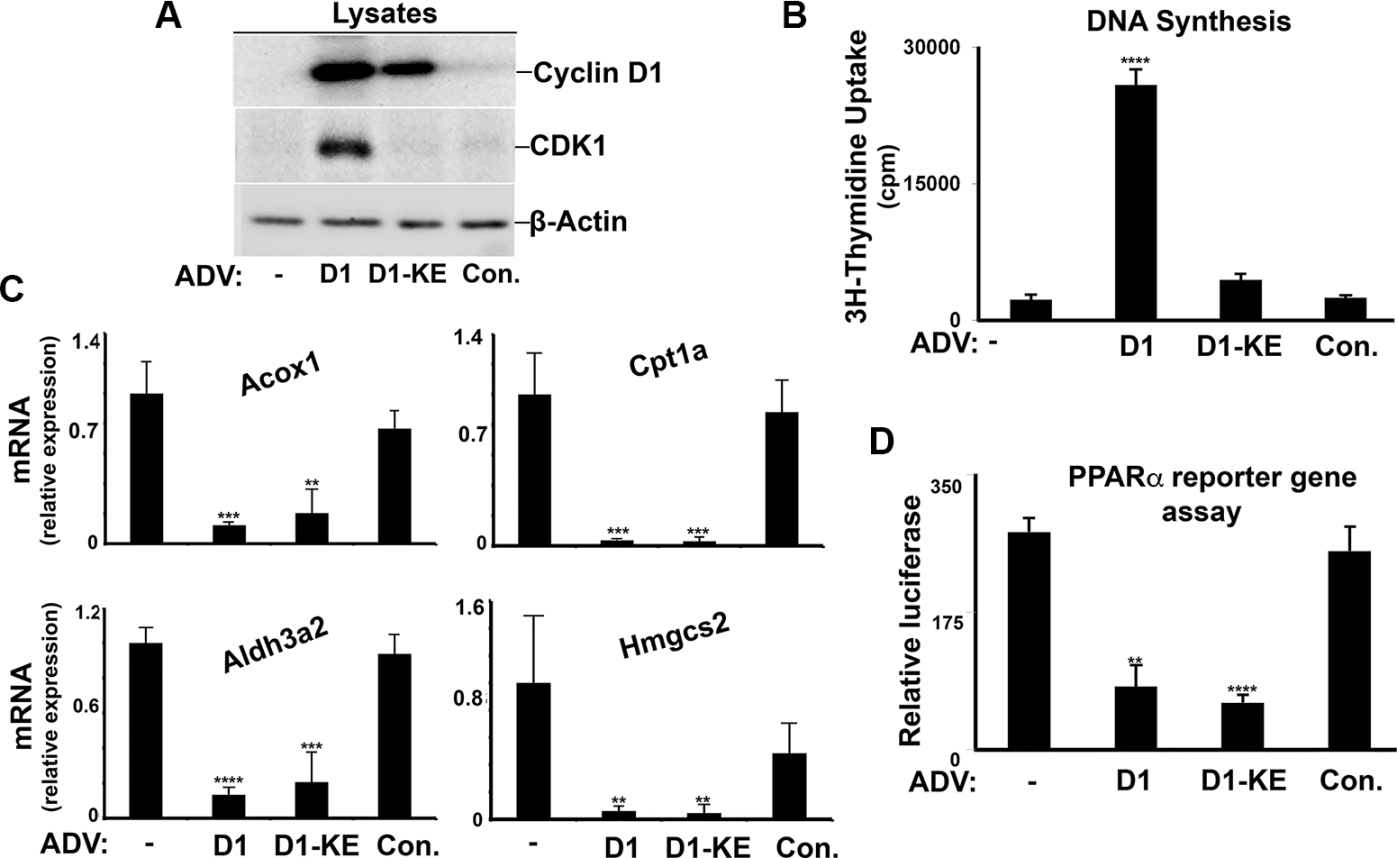
Cyclin D1 inhibits PPARα in hepatocytes Primary rat hepatocytes were cultured as previously described [18], and transduced with adenoviral vectors (ADV) encoding cyclins D1 or D1-KE or a control vector. Cells were harvested 2 days after adenoviral transduction. (**A**) Western blot analysis of the indicated proteins in cell lysates. (**B**) DNA synthesis. (**C**) Expression of representative PPARα-responsive transcripts by RT-PCR. All values normalized to Rpl32. (**D**) Transcriptional activity of transfected PPARα in a reporter gene assay.

Although the predominant cell type in the liver is the hepatocyte, many other cell types are present that could affect analysis of mRNA derived from whole liver [26]. We therefore isolated primary rat hepatocytes to initially examine the regulation of PPARα by cyclin D1 in these cells. Hepatocytes were cultured at high density (which promotes differentiated function) in the presence of a relatively low concentration of glucose (5.5 mM) [18], which activates the expression of PPARα target genes. The hepatocytes were transduced with wild-type cyclin D1 or a point mutant (K112E) that does not activate cdk4 (cyclin D1-KE) [27, 28], using adenoviral vectors which effectively target hepatocytes in culture and *in vivo*. As previously shown [8, 18], cyclin D1 (but not cyclin D1-KE) induces cell cycle progression in the absence of mitogenic stimuli, as evidenced by expression of a downstream cell cycle protein (cdk1) and DNA synthesis (Figure 1A–1B). Both cyclins D1 and D1-KE inhibited the expression of known PPARα target genes in hepatocytes cultured under low-glucose conditions (Figure 1C), including those involved in FA oxidation (carnitine palmitoyl transferase 1a [Cpt1a] and acyl-CoA oxidase [Acox 1]), aldehyde oxidation (aldehyde dehydrogenase 3 family, member A2 [Aldh3a2]), and ketogenesis (hydroxymethylglutaryl-CoA synthase 2 [Hmgcs2]). To examine whether cyclin D1 regulated PPARα, we employed an established assay using a PPARα construct fused to the DNA-binding domain of Gal4 along with a plasmid containing a Gal4-responsive promoter linked to luciferase. As shown in Figure 1D, both cyclin D1 variants inhibited the transcriptional activity of transfected PPARα in this assay. These data indicate that cyclin D1 inhibits PPARα transcriptional activity and target gene expression in a cdk4-independent manner.

**Figure 2:**
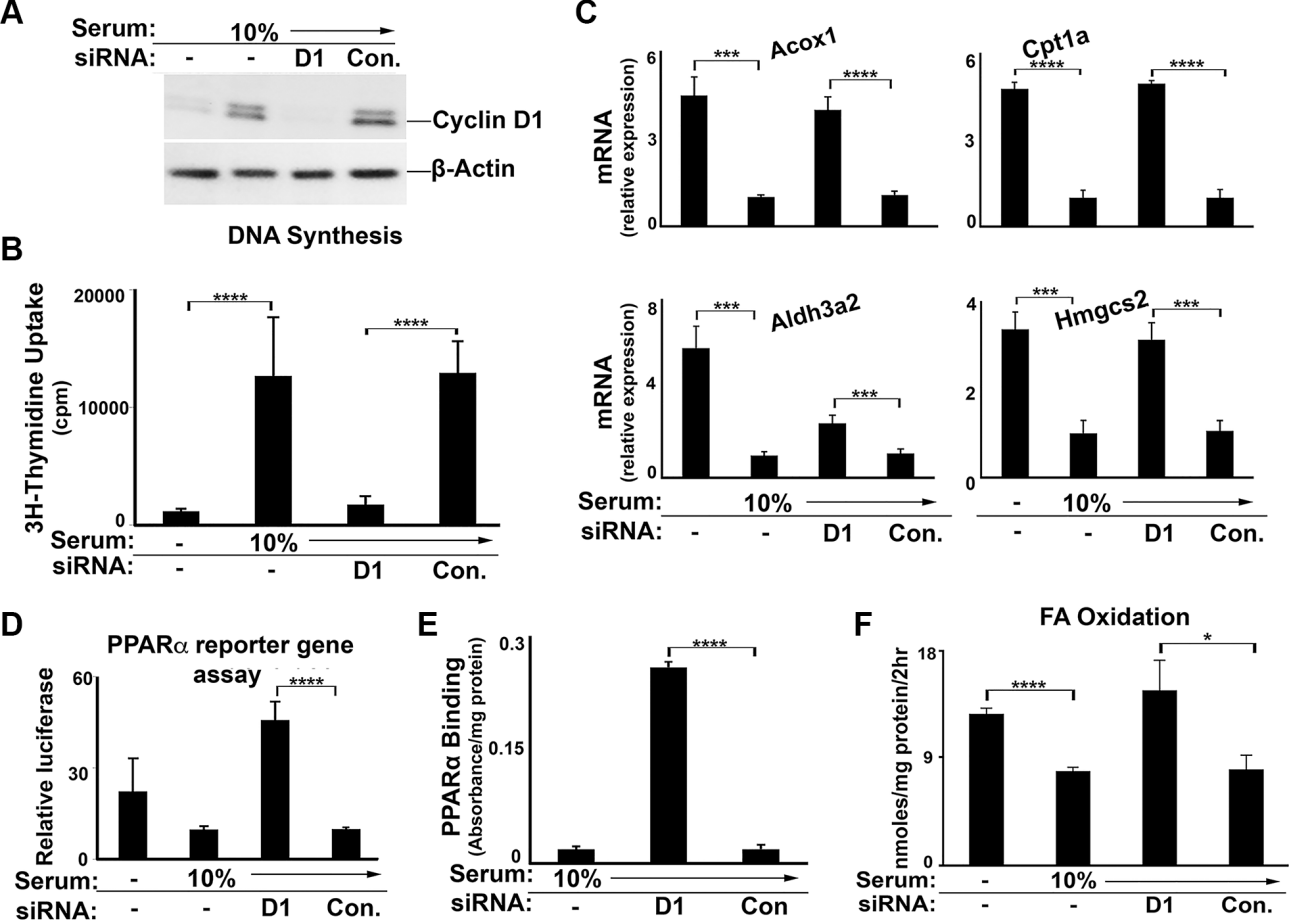
Cyclin D1 knockdown promotes PPARα activity and fatty acid oxidation in AML12 cells Cells were cultured in the presence or absence of 10% serum and cyclin D1 (D1) or control siRNA as indicated, and harvested after 48 hr. (**A**) Western blot. (**B**) DNA synthesis. (**C**) PPARα-mediated transcript expression. All values normalized to Gapdh. (**D**) PPARα transcriptional activity using the luciferase reporter system. (**E**) Binding of endogenous PPARα to a canonical DNA binding element by ELISA. (**F**) Fatty acid oxidation.

To examine the potential regulation of PPARα by endogenous cyclin D1, we first utilized the well-differentiated AML12 mouse hepatocyte cell line [29, 30]. Small interfering (si) RNA-mediated knockdown of cyclin D1 efficiently depleted the expression of this protein and inhibited serum-stimulated proliferation of these cells (Figure 2A–2B) [18]. Mitogenic stimulation with serum led to downregulation of PPARα-mediated gene expression (Figure 2C), which was largely reversed when cyclin D1 expression was depleted by siRNA. Knockdown of cyclin D1 increased the transcriptional activity of transfected PPARα in a reporter gene assay (Figure 2D), and enhanced the binding of endogenous PPARα to a consensus DNA motif in an *in vitro* assay (Figure 2E). In a similar manner, inhibition of cyclin D1 expression led to increased FA oxidation (Figure 2F). These studies demonstrate that mitogenic stimulation reduces PPARα activity and FA oxidation in a hepatic cell line, and that these effects are reversed by the depletion of cyclin D1. These results suggest that cyclin D1 is a pivotal mediator of both cell cycle progression and fatty acid oxidation in response to mitogenic stimuli.

**Figure 3:**
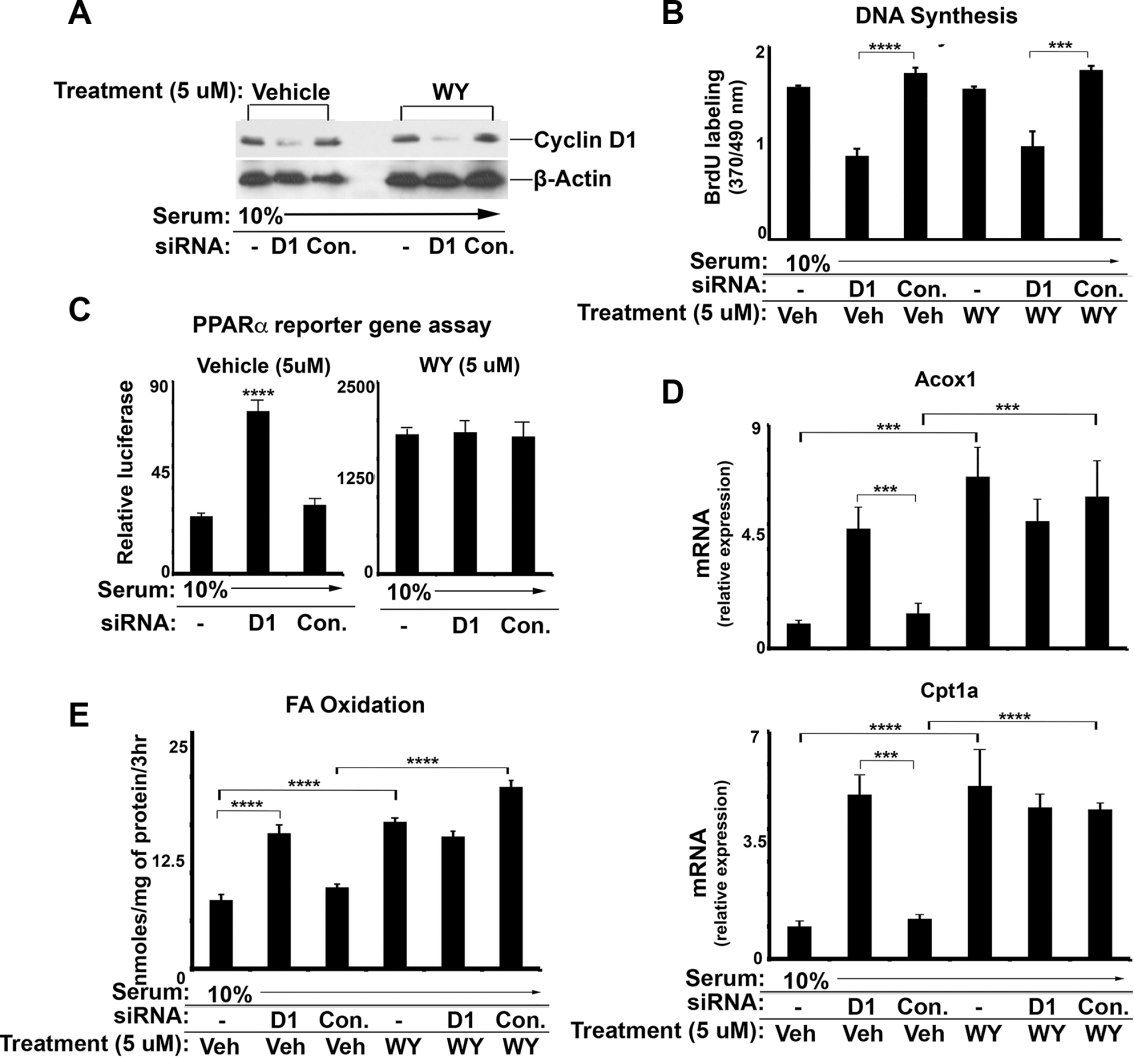
The effect of cyclin D1 knockdown and PPARa agonist treatment AML12 cells were cultured in the presence of serum (10%), subjected to siRNA transfection, and treated with 5 μM WY-14643 (WY) or vehicle (DMSO) as indicated. (**A**) Western blot. (**B**) DNA synthesis (**C**) PPARα transcriptional activity using the luciferase reporter system. (**D**) PPARα-mediated transcript expression by RT-PCR. (**E**) Fatty acid oxidation.

PPARα activity can be stimulated by treatment with pharmacologic ligands. In Figure 3, we treated AML12 cells with a potent PPARα ligand, WY-14643, which did not influence cyclin D1 expression (Figure 3A) or cell cycle progression as measured by DNA synthesis (Figure 3B). As expected, WY-14643 led to increased PPARα transcriptional activity as shown by reporter gene assay (Figure 3C), expression of PPARα target genes (Figure 3D), and FA oxidation (Figure 3E). Cyclin D1 siRNA upregulated each of these parameters in vehicle-treated cells. In cells treated with WY-14643, cyclin D1 knockdown did not further enhance PPARα activity or FA oxidation. Furthermore, the data in Figure 3D and 3E also indicate that cyclin D1 depletion in vehicle-treated cells stimulated target gene expression and FA oxidation to a similar extent as WY-14643 treatment. These findings suggest that cyclin D1 may regulate PPARα and FA oxidation under “physiologic” conditions, whereas pharmacologic activation of PPARα overwhelms the effect of cyclin D1. Furthermore, the effect of cyclin D1 knockdown on PPARα target genes and FA oxidation is similar in magnitude to ligand activation under the conditions studied here.

**Figure 4:**
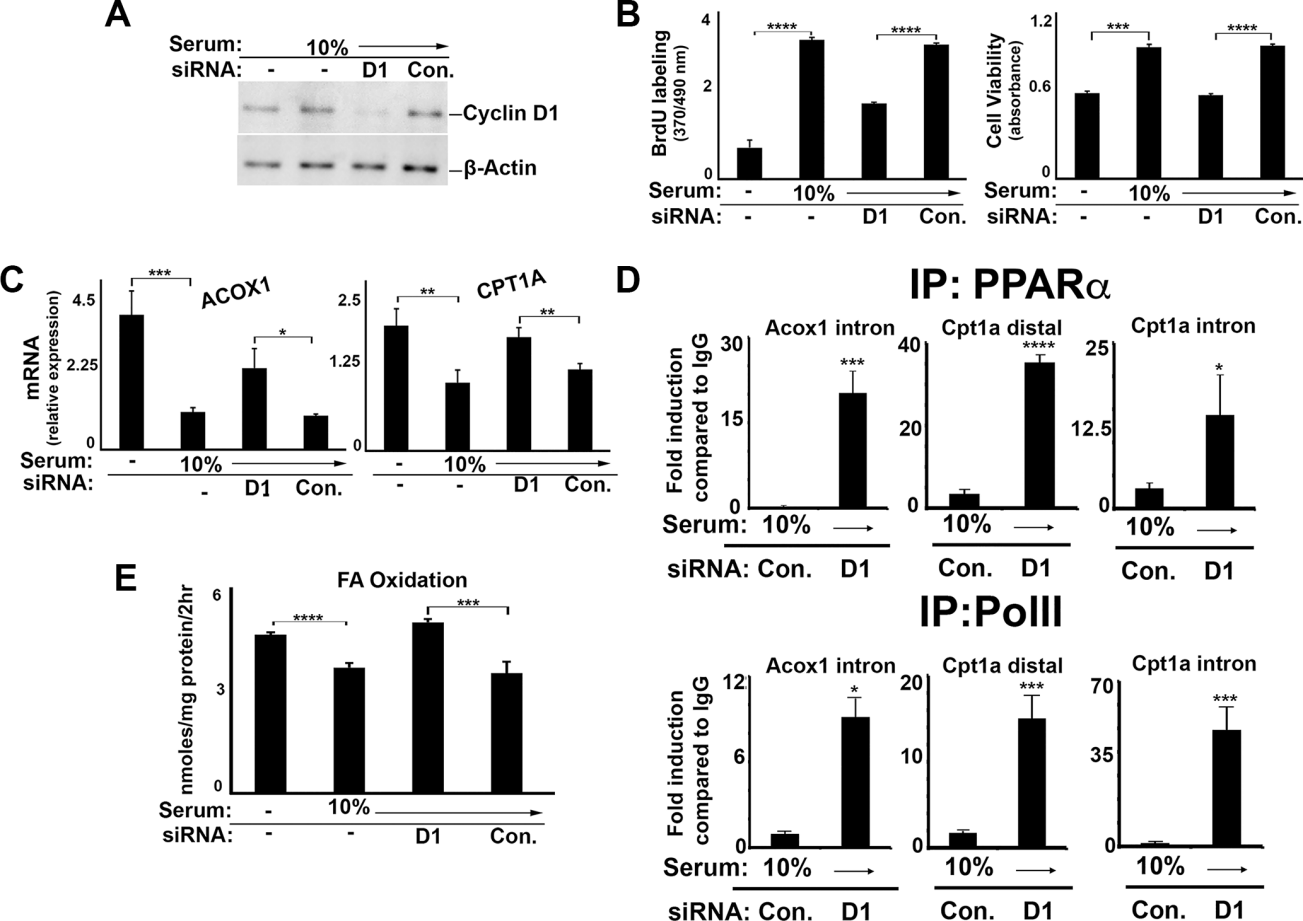
Cyclin D1 inhibits PPARα and fatty acid oxidation in liver cancer cells HepG2 cells were cultured in the presence or absence of serum and siRNA as indicted, and harvested after 72 hr. (**A**) Western blot. (**B**) DNA synthesis and cell viability (**C**) PPARα-mediated transcript expression. (**D**) Chromatin Immunoprecipitation (ChIP) using antibodies to PPARa (top) and PolII (bottom). (**E**) Fatty acid oxidation.

**Figure 5:**
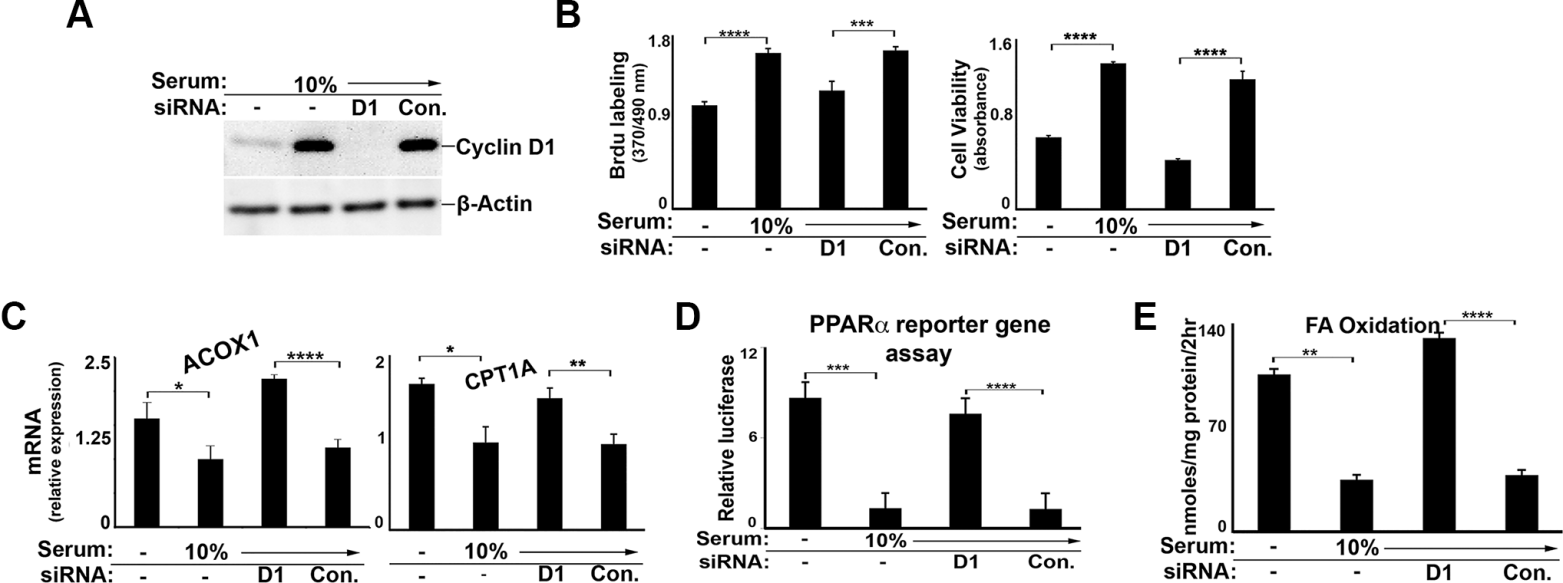
Cyclin D1 inhibits PPARα and fatty acid oxidation in breast cancer cells MCF7 cells were cultured in the presence of serum and siRNA as indicated. (**A**) Western blot. (**B**) DNA synthesis and cell viability (**C**) PPARα-mediated transcript expression. (**D**) PPARα luciferase reporter activity. (**E**) Fatty acid oxidation.

To examine whether cyclin D1 may regulate PPARα-mediated metabolism in malignant cells, we tested a number of different cancer cell lines. Cyclin D1 expression was efficiently depleted by siRNA in cells derived from liver cancer (HepG2) (Figure 4A) and breast cancer (MCF7) (Figure 5A), which resulted in decreased DNA synthesis and cell proliferation (Figure 4B and 5B). Similar to AML12 cells, serum stimulation led to decreased PPARα target gene expression (Figure 4C and 5C), the activity of transfected PPARα (Figure 5D), and FA oxidation (Figure 4E and 5E). In each case, knockdown of cyclin D1 led to a significant increase in these parameters, essentially counteracting the effect of serum stimulation. Furthermore, chromatin immunoprecipitation (ChIP) of PPARα in HepG2 cells demonstrated increased binding of this transcription factor to known target genes (CPT1A and ACOX1) in cyclin D1-depleted cells (Figure 4D). Cyclin D1 knockdown also led to increased binding of the RNA polymerase II at the same regions, suggesting transcriptional activation of these genes. Similar findings were observed in other human hepatocellular carcinoma (HCC) cell lines (HuH7 and SK-Hep1) (Supplementary Figure S2). Thus, cyclin D1 represses PPARα activity and FA oxidation in several different malignant cell lines.

**Figure 6:**
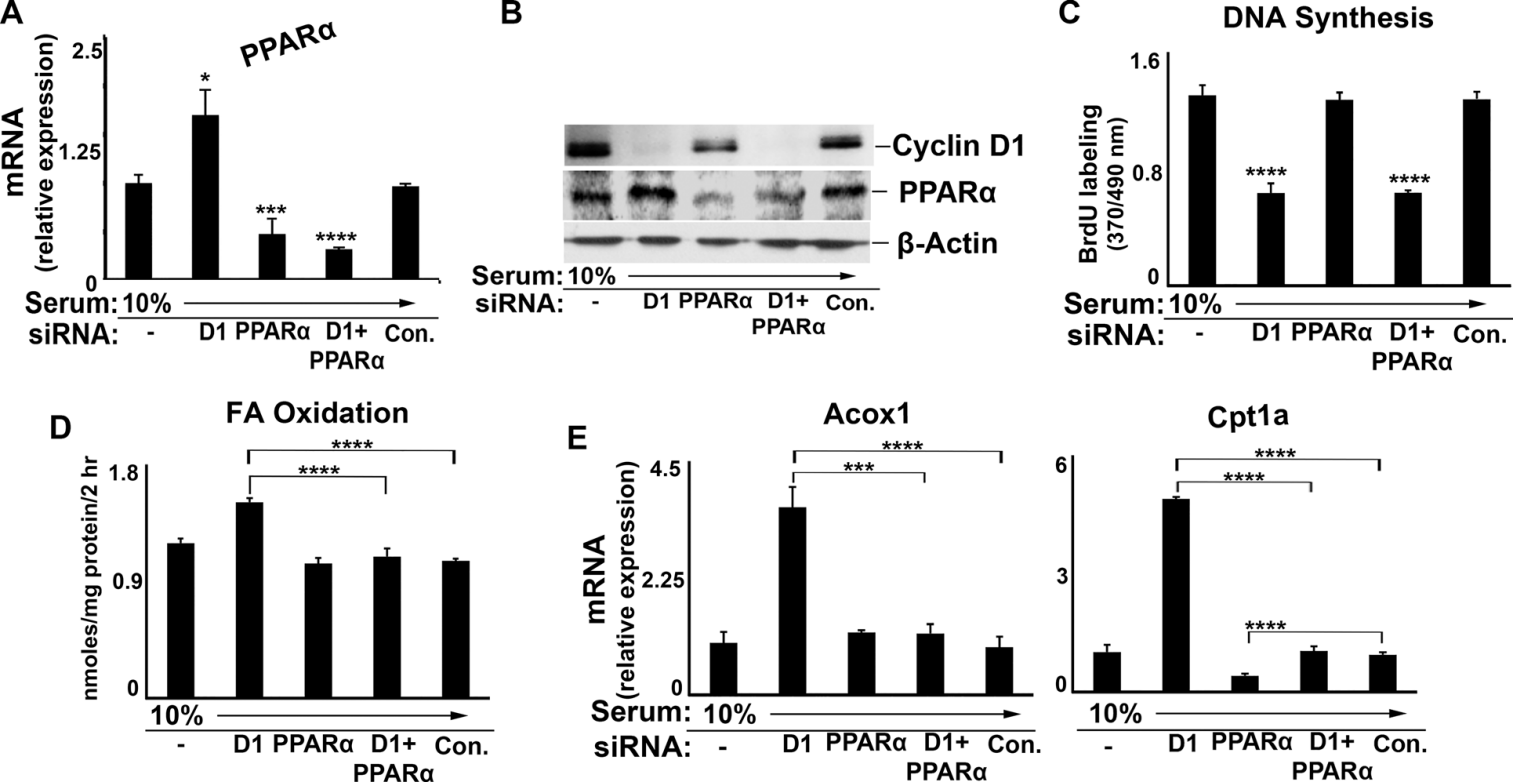
Effect of combined cyclin D1 and PPARα knockdown AML12 cells were cultured in the presence of serum and siRNA as indicated. (**A**) RT-PCR for PPARα. (**B**) Western blot. (**C**) DNA synthesis. (**D**) Fatty acid oxidation. (**E**) PPARα target gene expression.

Prior studies have found that cyclin D1 can affect gene expression by modulating the activity of numerous transcription factors and co-regulators [16–20]. To further establish that PPARα itself is a relevant target of cyclin D1, we examined the effect of PPARα knockdown in this system (Figure 6). siRNA-mediated depletion of PPARα reduced the expression of this protein by ~70% (Figure 6A and 6B), but had no effect on cellular proliferation (Figure 6C). The knockdown of PPARα modestly decreased Cpt1a expression and did not affect Acox1 expression in serum-stimulated AML12 cells (Figure 6E). This supports the observation that PPARα activity is already suppressed under conditions of mitogen stimulation (as shown in Figures 3–5). Consistent with this, depletion of PPARα did not further reduce FA oxidation in serum-stimulated cells (Figure 6D) As shown in the prior figures, cyclin D1 siRNA markedly increased expression of PPARα target genes and FA oxidation. However, when PPARα was also depleted, knockdown of cyclin D1 had little effect on target gene expression (Figure 6E) or FA oxidation (Figure 6D). Similar findings were noted in the HepG2, HuH7 and MCF7 cell lines (Supplementary Figure S3). These findings strongly support the concept that cyclin D1 regulates the expression of these genes and FA oxidation, at least in part, through modulation of PPARα activity.

**Figure 7:**
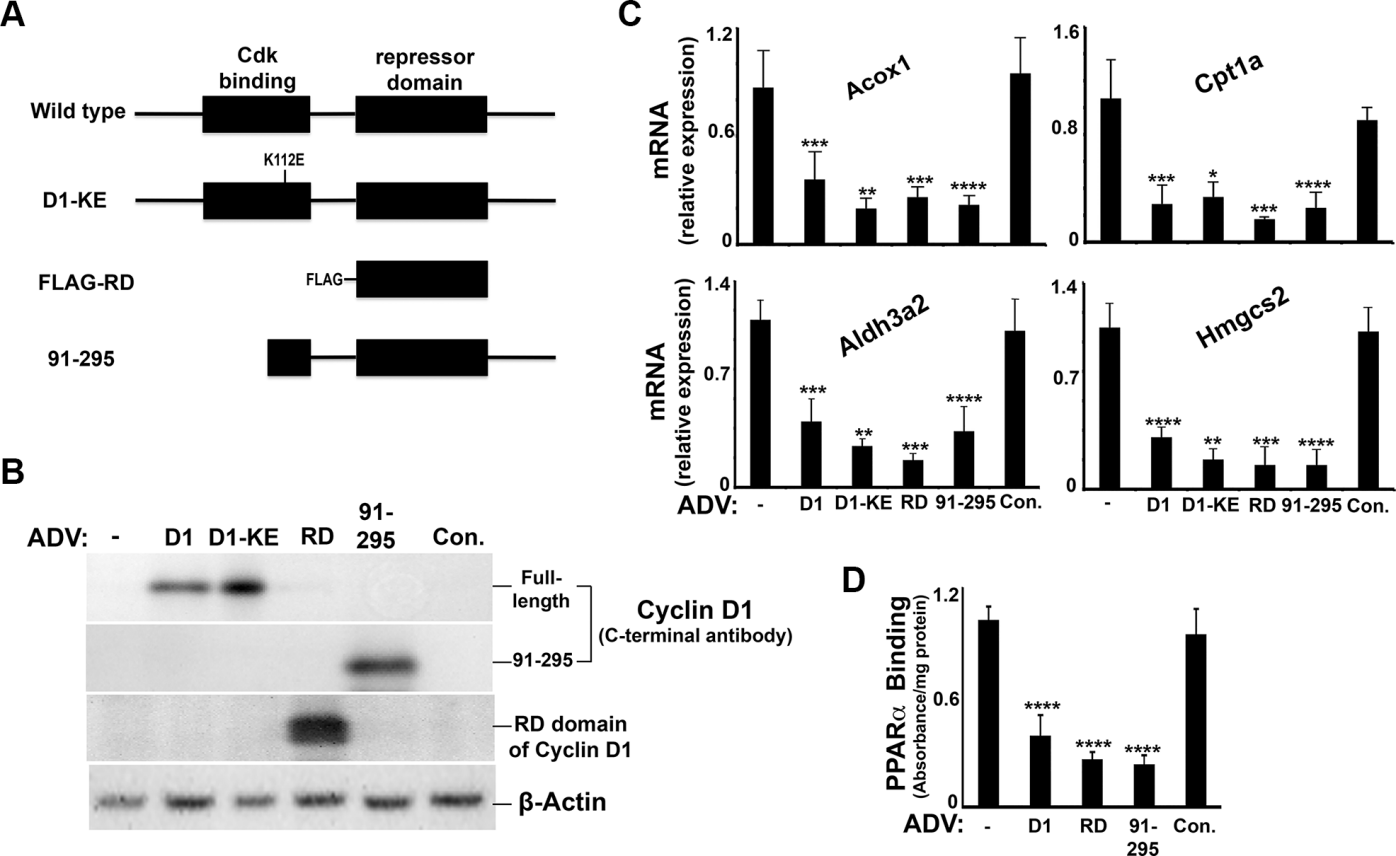
Cyclin D1 represses PPARα activity in the liver Mice were transduced with adenoviral vectors encoding cyclin D1 (and the indicated mutant forms) or a control vector and livers harvested after 48 hours. (**A**) Diagram of cyclin D1 mutants used. (**B**) Western blot. (**C**) Expression of PPARα-regulated transcripts. (**D**) Binding of endogenous PPARα to a target sequence by ELISA.

Previous studies have shown that a central portion of the cyclin D1 protein, termed the “repressor domain” (RD)(amino acids 141–250), inhibits the activity of the nuclear receptors HNF4α and androgen receptor [16, 18]. In Figure 7, we transduced livers *in vivo* with cyclin D1 variants including the RD domain alone and an N-terminal truncation mutant of cyclin D1 (91–295), which do not contain the region that binds cdk4 [16, 31] (Figure 7A). Each of these constructs successfully expressed in the liver (Figure 7B), significantly repressed the expression of PPARα target genes in the liver (Figure 7C), and led to decreased PPARα DNA binding activity in an *in vitro* assay (Figure 7D). These data suggest that cyclin D1 regulates PPARα activity *in vivo* via the RD domain and does not require cdk4.

**Figure 8:**
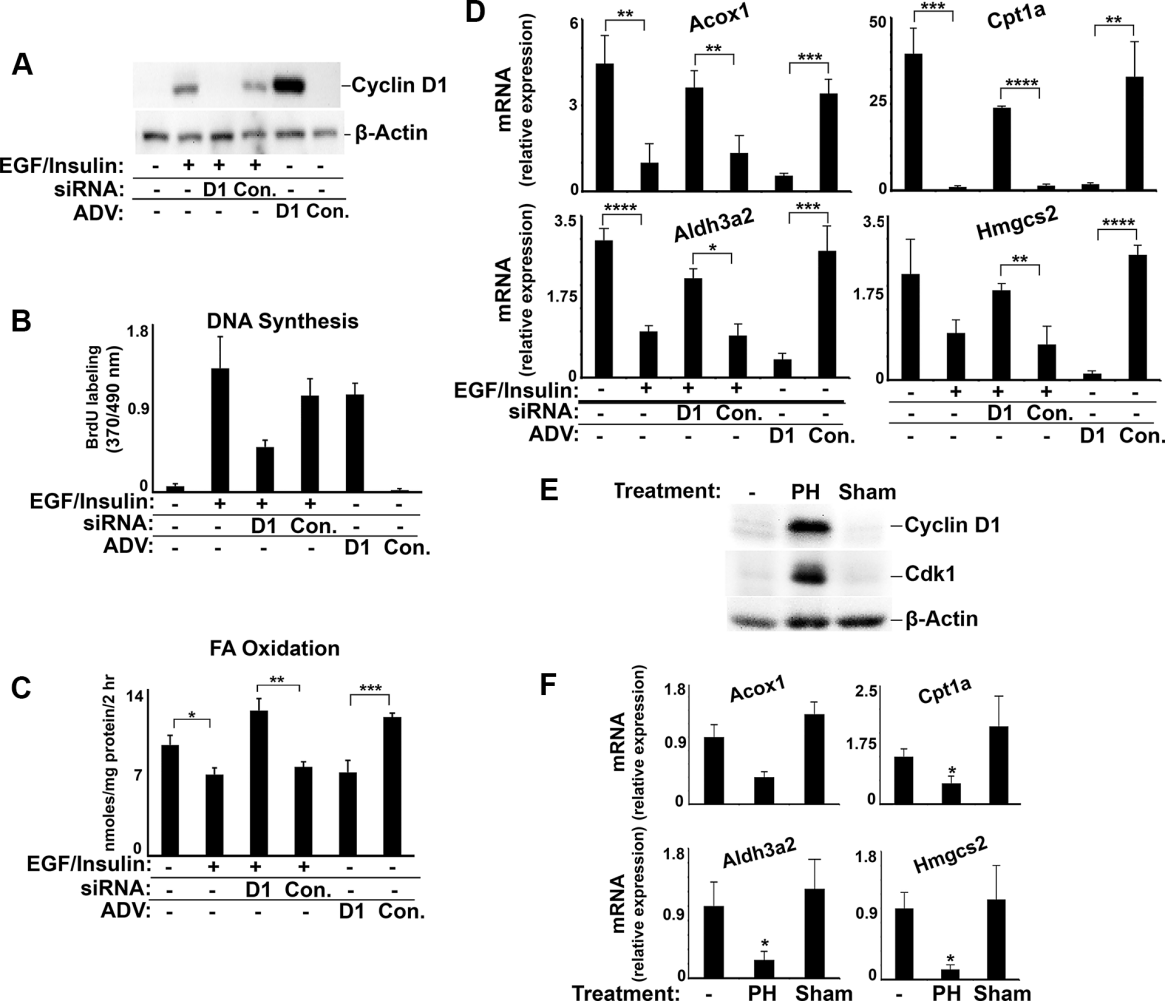
Regulation of PPARα-mediated gene and FA oxidation in proliferating hepatocytes and regenerating liver Hepatocytes were cultured in the presence or absence of mitogens (EGF with insulin) [8], treated with siRNA or adenovirus as indicated (**A**–**D**), and harvested 3 days later. 70% PH or sham surgery were performed male mice and livers harvested at 42 hours (**E**–**F**). (A) Western blot. (B) DNA synthesis. (C) Fatty acid oxidation. (D) Expression of PPARα-mediated transcripts. (E) Western blot of lysates from mouse liver. (F) Expression of PPARα-mediated genes in mouse liver.

We next examined whether hepatic PPARα activity might be inhibited by endogenous cyclin D1 during hepatocyte proliferation. In Figure 8, we cultured hepatocytes under conditions that promote growth factor-mediated cell cycle progression [8]. Mitogen stimulation led to induction of cyclin D1 and robust proliferation of these cells, which was significantly inhibited by cyclin D1 siRNA (Figure 8A–8B). Conversely, transduction of mitogen-deprived hepatocytes with cyclin D1 stimulated marked proliferation [8]. FA oxidation was decreased by mitogen stimulation, and this effect was reversed by the knockdown of cyclin D1 (Figure 8C). On the other hand, expression of cyclin D1 in growth factor-deprived cells was sufficient to reduce FA oxidation to the levels seen after mitogen stimulation. Similar changes were seen in the expression of PPARα-regulated genes involved in FA oxidation (Figure 8D). Following 70% partial hepatectomy in mice, the standard model of hepatocyte proliferation *in vivo*, induction of endogenous cyclin D1 was associated with decreased expression of these same genes (Figure 8E–8F), which is consistent with the concept that cyclin D1 represses PPARα activity in the regenerating liver.

## DISCUSSION

Recent literature has established that several key oncogenes play a role in metabolic reprogramming to allow for cancer cell growth and proliferation [1–4]. Since normal cell proliferation is controlled by many of the same drivers of cancer cell proliferation, similar metabolic adaptations can be observed. Much of the literature on cancer metabolism has focused on increased glucose and glutamine utilization, but the full spectrum of alterations seen in growth and proliferation remains to be defined. The data presented here demonstrate that cyclin D1, an important cell cycle control protein and proto- oncogene, inhibits PPARα-mediated gene expression and FA oxidation in both hepatocytes and cell lines derived from hepatocellular and breast cancer. These studies offer further insight into the complex interplay between cell cycle control, growth, and metabolism in normal and malignant cells.

Substantial metabolic adaptations occur during cell proliferation, but the mechanisms that link the cell cycle machinery to metabolic regulators have remained largely obscure [4, 32]. Cyclin D1 associates with the promoters of hundreds of genes *in vivo*, some of which encode metabolic genes, suggesting that it may play a broad role in regulating metabolism during proliferation [14]. Previous studies have shown that cyclin D1 regulates nuclear receptors including the androgen and estrogen receptors, PPARγ, thyroid hormone receptor β, and HNF4α through several distinct mechanisms [16–20], and thus can regulate metabolism in various cell types. We have recently shown that cyclin D1 represses HNF4α transcriptional activity in hepatocytes [18], leading to downregulation of *de novo* lipogenesis. HNF4α regulates many differentiated functions in hepatocytes (including lipogenesis) [33], but does not play a role in most other tissues. On the other hand, PPARα is expressed in many different organs and cell types, and thus its regulation by cyclin D1 may be a more generalized feature in proliferating cells.

We present several lines of evidence that cyclin D1 regulates cell metabolism via inhibition of PPARα activity. First, cyclin D1 inhibits PPARα transcriptional activity, target gene expression, and fatty acid oxidation in hepatocytes (Figures 1 and 8). Conversely, knockdown of cyclin D1 stimulates these parameters in hepatocytes and cancer cell lines (Figures 2, 4, 5, and 8). Upon depletion of PPARα, these effects of cyclin D1 knockdown are markedly attenuated, (Figure 6 and Supplementary Figure S3), strongly suggesting that cyclin D1's effect on FA oxidation is mediated, at least in part, by PPARα. Cyclin D1 appears to regulate PPARα activity in a cdk4-independent manner (Figure 1 and 7). In mitogen-stimulated hepatocytes in culture and in regenerating liver *in vivo*, induction of cyclin D1 correlates with diminished PPARα-mediated gene expression (Figure 8). Because numerous regulatory pathways modulate FA oxidation, we cannot rule out the possibility that cyclin D1 affects FA oxidation and gene expression through other mediators in addition to PPARα. However, in both hepatocytes and the cancer cell lines examined here, cyclin D1 represses PPARα activity and FA oxidation.

Compared to glucose and glutamine metabolism, the role of fatty acid oxidation and PPARα activity in proliferating and malignant cells has received relatively little attention [34, 35]. In hepatocytes, AML12 cells, and the cancer cell lines used in our studies, mitogenic stimulation led to decreased PPARα activity and FA oxidation. This suggests that part of the metabolic “switch” that occurs during the transition from quiescence to proliferation involves decreased energy production from fatty acids. Similar findings have been reported in activated lymphocytes [36, 37]. Although FA oxidation is often down-regulated in proliferating cancer cells, these cells may retain significant metabolic flexibility, and can utilize fatty acids for fuel sources as an adaptive response to environmental stresses [2, 3, 34]. Our data indicate that cyclin D1 plays a pivotal role in inhibiting FA oxidation in response to mitogens.

Previous studies have suggested that activation of PPARα can have anti-tumor effects [35, 38]. Although it is present in highest abundance in organs involved in lipid catabolism (such as the liver), PPARα is detectable in a variety of cancer cell types. PPARα agonist drugs inhibit proliferation, decrease invasiveness, and/or trigger apoptosis in cancer cell lines derived from breast, endometrial, lymphatic, central nervous system, and other malignancies [39–43]. Older studies had shown that long-term exposure to PPARα agonists in rodents led to HCC, but this was likely due to chronic oxidative stress and resulting inflammation rather than a direct effect of PPARα [44]. Indeed, PPARα knockout mice are more susceptible to carcinogen-induced HCC, suggesting that PPARα inhibits tumor formation in this model [45]. Furthermore, the PPARα agonist fenofibrate suppresses tumor growth in mice via decreased angiogenesis [46]. The mechanisms underlying the antineoplastic effects of PPARα have not been clearly elucidated, but presumably include altered cell metabolism and energetics [35, 38]. The findings presented here suggest that by repressing the activity of PPARα, cyclin D1 could inhibit the antineoplastic effect(s) of this nuclear receptor.

It has long been recognized that triglycerides, fatty acids, and other lipids accumulate in the regenerating liver after PH, which is associated morphologically with increased hepatocyte lipid droplets. The timing of hepatic accumulation of lipid droplets roughly corresponds to the induction of cyclin D1 in this model [47–49]. The mechanisms that drive hepatic fat accumulation are complex and likely to be modulated by numerous physiologic factors, given the liver's pivotal role in systemic lipid metabolism. In the current study, we show that PPARα-mediated gene expression is down-regulated in regenerating liver after PH (corresponding to enhanced cyclin D1 expression, Figure 8), as reported previously [50], and that transduction of cyclin D1 into hepatocytes or the liver produces similar effects (Figure 7–8). Moreover, knockdown of cyclin D1 prevents the inhibition of PPARα target genes and FA oxidation by mitogen stimulation in hepatocytes, while cyclin D1 transduction into mitogen-deprived cells is sufficient to inhibit these effects (Figure 8). These studies suggest that cyclin D1 expression is both necessary and sufficient to inhibit PPARα-mediated gene expression and FA oxidation under these conditions. Older studies have shown that ablation of PPARα promotes fatty liver [51]. The data presented here suggest that inhibition of PPARα activity and FA oxidation by cyclin D1 may contribute to hepatic lipid accumulation during liver regeneration.

The mechanisms that regulate PPARα activity are complex and incompletely characterized [24, 51, 52]. Our data suggest that cyclin D1 diminishes binding of PPARα to target sequences, which has also been shown for the related nuclear receptors PPARγ and androgen receptor [19, 53]. However, given the complexity of PPARα regulation, the detailed mechanism(s) by which cyclin D1 regulates its activity remain to be worked out. For example, in addition to regulating binding of PPARα to target sequences, cyclin D1 could affect the numerous co-regulatory proteins that modulate PPARα-mediated gene expression after it is bound to chromatin. In addition, it is conceivable that cyclin D1 regulates the abundance of physiologic PPARα ligands within the cell; prior studies have shown that it significantly affects androgen and estrogen levels in the liver [54], which are ligands for distinct nuclear receptors. In AML12 cells, cyclin D1 knockdown had similar effects to ligand activation (Figure 3), which is consistent with the possibility that cyclin D1 may regulate ligand availability. Furthermore, PPARα is regulated by post-translational modifications and localization within the cell. Additional study will be required to examine each of these mechanisms, and to determine whether other PPARα-mediated processes including inflammation, drug metabolism, and angiogenesis are also regulated by cyclin D1.

In summary, cyclin D1 inhibits PPARα activity and FA oxidation in response to mitogenic signaling, and thus regulates a key aspect of energy metabolism during proliferation. These studies provide further insight into the role of cyclin D1 beyond activation of the cell cycle machinery. Given the current interest in identifying potential metabolic targets in cancer [1–5, 34], further study of the cyclin D1-PPARα regulatory interaction may provide insight into rationally-designed treatment strategies.

## MATERIALS AND METHODS

### Animals

All animals were housed according to National Institute of Health Guidelines. Experiments were carried out under the supervision of the Institutional Animal Care and Use Committee at Minneapolis Medical Research Foundation. Young male Sprague Dawley rats (225–250 gm) and 8–10 week old male Balb/C mice were purchased from Harlan Sprague Dawley. Mice were subjected to 70% partial hepatectomy (PH), sham surgery, and adenoviral transduction using 5–7 × 10^9^ plaque forming units (pfu)/animal, as previously described [10]. Sham surgeries were performed similarly to PH, including externalization of the liver. Four to six animals were used for each condition and time point.

### Primary hepatocytes

Rat hepatocytes were obtained via a two-step collagenase perfusion method as previously described [8]. In Figure 1, these cells were cultured under conditions that promote differentiated function [18], and harvested 2 days after plating. In Figure 6, cells were cultured under conditions that promote mitogen-induced proliferation [8], and harvested 3 days after plating. Adenovirus transduction was performed as previously described [8, 18]. On-Target plus SMARTpool siRNA directed against rat cyclin D1 (catalog # L-089285-02-0020,) and matching non-specific siRNA (catalog # D-001810-10-20) were used at 2 nM along with DharmaFECT (catalog # T-2004-02) according to the manufacturer's instructions (Dharmacon, Lafayette, CO).

### Cell lines

AML12 cells and HuH7 were cultured as previously described [18, 54]. MCF-7 cells (a gift of Dr. Carol Lange, University of Minnesota) and HepG2 (Catalog #ATCC^®^ HB-8065^™^, ATCC, Manassas, VA) cells were cultured in DMEM media in 10% fetal bovine serum. On-Target plus SMARTpool siRNA directed against human (catalog # L-003210-00-0020) or mouse cyclin D1 (catalog # L-042441-00-0020) and human (catalog # L-003434-00-0020) or mouse PPARα (catalog # L-040740-01-0020) from Dharmacon, Lafayette, CO, were used as above. After 24 hours, fresh media was added and at 48 hours cells were harvested for different assays. Serum was withdrawn for the final 24 hours in selected conditions as shown in the figures. In Figure 3, cells were treated with 5 mM WY-14643 (Catalog # 70730, Cayman, Ann Arbor, MI) or DMSO vehicle for 24 hours before harvest.

### Adenoviruses

Adenovirus vectors for cyclins D1, D1-KE, and D1-RD have been described previously [18, 54]. The adenovirus encoding the N-terminal truncation mutant encoding amino acids 91–295 of cyclin D1 was constructed by Vector Biolabs, using cDNA provided by Dr. Rolf Muller [31]. For control experiments, an adenovirus expressing GFP was purchased from Vector Biolabs (Catalog #1060, Vector Biolabs, Malvern, PA.).

### DNA synthesis and Cell Viability

DNA synthesis was measured using ^3^H thymidine incorporation as previously described [8], or using an ELISA BrdU labeling kit (Catalog #11647229001, Roche Diagnostics, Indianapolis, IN) following manufacturer's protocol. Cell viability was measured using the CellTiter-Blue^®^ Cell Viability assay kit (Catalog # G8080, Promega, Madison, WI) as instructed by the manufacturer.

### Protein extraction and western blotting

These procedures were performed as previously described [10, 18]. Anti-Cyclin D1 antibody (Catalog #04-221) was from Millipore, (Billerica, MA), anti-CDK1 antibody (Catalog# SC-54) and anti-PPARa antibody (SC-9000) were from Santa Cruz Biotechnology (Dallas, TX). Anti-Actin antibody (Catalog #A2066) was purchased from Sigma (St.Louis, MO). The secondary antibodies anti-rabbit (Catalog # 7050) and anti-mouse (Catalog #7056) conjugated with alkaline phosphatase were purchased from Cell Signaling (Boston, MA).

### RNA isolation and real time RT-PCR

These procedures were performed as previously described [18]. Primer sequences are in Supplementary Table S1. RT-PCR results were normalized to Rpl32 in primary hepatocytes and to GAPDH in cell lines and mouse liver.

### Fatty acid oxidation

Cells were labeled with 500 μM of oleate containing 1 μCi of [1-^14^C] oleate for 2 hours. Fatty acid oxidation was measured by following radiolabeled oleate metabolism into acid-soluble metabolites (ASM). Briefly, 200 μL of perchloric acid was added to 900 μL of media followed by centrifugation (10,000 × g for 15 min). 500 μL of the supernatant was combined with 4 mL of scintillation cocktail and was quantified by scintillation counting.

### PPARa transcription factor binding assays

1 gm of mouse liver tissue or 6 million cultured cells were homogenized using a nuclear extraction kit protocol (Catalog # 10009277, Cayman Chemical, Ann Arbor, MI). We followed the manufacturer's protocol for nuclear extraction. 10 mL of nuclear extract was used for a PPARa transcription factor-binding assay (Catalog #10006915, Cayman Chemical, Ann Arbor, MI) and the readings were normalized to total nuclear protein extracts according to the manufacturer's instruction. This assay measures endogenous PPARα activity in nuclear lysates by measuring the binding of this protein to a peroxisome proliferator response DNA elements immobilized on 96-well plates, which is followed by ELISA for PPARα using anti-PPARa antibody provided by the kit.

### PPARα reporter gene assays

PPARα promoter luciferase assays were performed using 200 ng of TK-MH-UAS-LUC luciferase reporter plasmid, 25 ng of pSG5-GAL4-hPPARα expression plasmid, and 25 ng of pRL-SV40 renilla plasmid along with Lipofectamine^®^ LTX Reagent with Plus^™^ Reagent (Catalog # 15338100, ThermoFisher, Grand Island, NY) per well on a 24 well plate in triplicates, as previously described [55]. The GAL4-PPARα chimeric construct used in these studies contains the ligand-binding domain of human PPARα, and when activated it promotes luciferase activity from a GAL4-responsive luciferase vector. Thus, this assay measures activity from the transfected PPARα construct rather than endogenous PPARα.

### Chromatin Immunoprecipitation (ChIP)

3.5 million of HepG2 cells were cultured in 10-cm plates and transfected with cyclin D1 and control siRNAs respectively. After 72 hours post-transfection, cells were crosslinked by adding formaldehyde to a final concentration of 0.4% to the media directly and incubated for 10 min on a shaker at room temperature (RT). The crosslinking was stopped by adding 0.125 M freshly made glycine for 5 min at RT. Cells were washed with ice-cold PBS for three times and lysed in 1 mL of cell lysis buffer (5 mM PIPES pH 8.0, 85 mM KCl, 0.5% IGEPAL CA-630, and protease inhibitors). The lysates were incubated on ice for 15 min, followed by centrifugation at 4000 RPM for 5 min at 4°C. The supernatant was discarded and the pellet was resuspended in 350 μL of SDS lysis buffer (10 mM EDTA, 50 mM Tris-Hcl pH 8.1, and 1% SDS) with protease inhibitors. The lysates were incubated on ice for 10 min followed by sonication at 40 W, 100%, (15 sec) X3 using Bioruptor^®^ sonicator device from Diagenode (Denville, NJ). After sonication, chromatin was centrifuged at 14,000 RPM for 20 min at 4°C. The chromatin supernatant was taken to perform chromatin immunoprecipitation using MAGnify™ ChiP kit (Catalog #492024, ThermoFisher Scientific, Grand Island, NY) following instructions as indicated in the manufacturer's protocol. Anti-PPARa antibody (Catalog #SC-9000) and anti-PolII antibody (Catalog # SC-900x) were used to immunoprecipate PPARa and PolII from the chromatin using DynaMag™-PCR Magnet (Catalog # 492025, ThermoFisher Scientific, Grand Island, NY). After reverse crosslinking, purified DNA samples were analyzed by real-time quantitative PCR. Final results represent ct values normalized to ct values of IgG. The ChIP primer sequences are in Supplementary Table S2.

### Statistical analysis

Data are expressed as mean ± standard error of mean. Statistical analysis was performed using GraphPad Software (GraphPad Software, Inc. La Jolla, CA). Comparisons between two groups were made by Student *t* test and difference among selected experimental groups with *p ≤* 0.05 were considered significant. *P* values *≤* 0.0005 represented as ****values *≤* 0.005 represented as ***values *≤* 0.01 represented as **and ≤ 0.05 represented as *.

## SUPPLEMENTARY MATERIALS



## Abbreviations

cdk: cyclin-dependent kinase
ChIP: chromatin immunoprecipitation
FA: fatty acid
HNF4α: hepatocyte nuclear factor 4α
PPAR: peroxisome proliferator-activated receptor
