# Supplementary material for: Cyclin D1 represses peroxisome proliferator-activated receptor alpha and inhibits fatty acid oxidation
Source: Oncotarget. 2016 Jun 24;7(30):47674–86. doi: 10.18632/oncotarget.10274 (PMC5216970; doi:10.18632/oncotarget.10274)
Supplement: Supplementary file 1 [file oncotarget-07-47674-s001.pdf]

Cyclin D1 represses peroxisome proliferator-activated receptor alpha and inhibits fatty acid oxidation

Supplementary Materials

MATERIALS AND METHODS

Identifying PPARα targets from array data

Potential downstream targets of PPARα were identified using Ingenuity Pathway Analysis (IPA). Of 198 possible PPARα targets from IPA, 56 were found in our previously published gene array from cyclin D1-transduced livers (1). They mean fold change of these genes relative to control-transduced livers was calculated (PPARMean). Fifty-six different genes were selected randomly from the array data and the mean fold change was calculated (RandMean). The difference between the PPARMean and the RandMean was calculated (DeltaMean). This process was repeated 10,000 times resulting in the distribution DeltaMeans.

If the downstream targets of Ppara showed gene expression changes that were similar to the randomly selected genes then the DeltaMean would be zero. The z-score for the PPARMean was calculated using equation 1 to understand how difference from 0 PPARMean was $z = \frac{(0 - mean(DeltaMeans))}{\sigma(DeltaMean)}$. A *p*-value was assigned to the z-score based on a normal distribution with a mean of 0 using the R version 3.2.3 command pnorm() (equation 2). $p = 2\left(pnorm(|z|)\right)$

Using IPA to identify downstream targets of PPARα

Starting with PPARα the Grow and Trim tools were used to identify downstream targets of PPARα. The following filters used with Grow Tool;

General settings

- direct connections
- max 300 molecules
- downstream of selected molecules
- use the Ingenuity Knowledge Base

Data sources

- Removed miRBase, miRecords, TarBase, TargetScan Human

Confidence level

- Experimentally Observed Only

Species

- Human and Mouse Only

No changes to the other options

Trim tool was used to remove mature microRNA and microRNAs.

Cell lines and transfection of siRNA

HuH7 were cultured as previously described (2). SK-Hep1 (ATCC® HTB-52™, ATCC, Manassas, VA) cells were cultured in DMEM media in 10% fetal bovine serum. Transfection of siRNAs, measurement of DNA synthesis, RNA isolation and RT-PCR, and fatty acid oxidation assays and are described in the main Material and Methods section.

REFERENCES

1. Mullany LK, White P, Hanse EA, Nelsen CJ, Goggin MM, Mullany JE, Anttila CK, et al. Distinct proliferative and transcriptional effects of the D-type cyclins in vivo. Cell Cycle 2008; 7:2215–2224.
2. Mullany LK, Hanse EA, Romano A, Blomquist CH, Mason JI, Delvoux B, Anttila C, et al. Cyclin D1 regulates hepatic estrogen and androgen metabolism. Am J Physiol Gastrointest Liver Physiol 2010; 298:G884–895.
3. Hu X, Li Y, Li C, Fu Y, Cai F, Chen Q, Li D. Combination of fucoxanthin and conjugated linoleic acid attenuates body weight gain and improves lipid metabolism in high-fat diet-induced obese rats. Arch Biochem Biophys 2012; 519:59–65.
4. Rondini EA, Bennink MR. Microarray Analyses of Genes Differentially Expressed by Diet (Black Beans and Soy Flour) during Azoxymethane-Induced Colon Carcinogenesis in Rats. J Nutr Metab 2012; 2012:351796.
5. Heinaniemi M, Uski JO, Degenhardt T, Carlberg C. Meta-analysis of primary target genes of peroxisome proliferator-activated receptors. Genome Biol 2007; 8:R147.

Supplementary Table S1:

Species	Gene	FWD (5′–3′)	REV (5′–3′)	Reference
Rat	Acox1	ACAGTTCTGAGAGCACAGCATC	CATTCCAGGAGAAAGGTTAAGGC	
Rat	Aldh3A2	TCTGAGGCAGCGGTTTGATC	CCAACTGCGGTGTTTCCTGT	
Rat	Cpt1a	CGCTCATGGTCAACAGCAACTAC	TCACGGTCTAATGTGCGACGA	(3)
Rat	Hmgcs2	TGCCCAAACGTCTAGACTCC	GGAGAGAAGTTCACCTTGTGGT	(4)
Rat	Rpl32	AAACTGGCGGAAACCCAGAG	GCAGCACTTCCAGCTCCTTG	
Mouse	Acox1	TTGTTGTCCCTATCCGTGAGA	GGCCGATATCCCCAACAGT	
Mouse	Aldh3A2	TCATGTACTTTCCCCAGGCT	AAGCATCTGACCCCTGTGAC	
Mouse	Cpt1a	CCCTGGGCATGATTGCAA	AAGAGGACGCCACTCACCAT	
Mouse	Hmgcs2	ATACCACCAACGCCTGTTATGG	CAATGTCACCACAGACCACCAG	
Mouse	PPARa	CTGCAGAGCAACCATCCAGAT	GCCGAAGGTCCACCATTTT	
Human	Acox1	CCAAGCTTTCCTGCTCAGTGTT	CCCCCAGTCCCTTTTCTTCA	
Human	Cpt1a	AGATTTTGCTGTCGGTCTTGGA	CACCAGTCGCTCACGTAATTTG	
Mouse/Human	Gapdh	GCATCCTGCACCACCA	GGATGACCTTGCCCACA	

Supplementary Table S2:

Species	Gene/region	FWD (5′–3′)	REV (5′–3′)	Reference
Human	Cpt1a-distal	GAAGCTGGACTGTCATTCTCAC	TGAGCGAGGGAGGAAATG	https://genome.ucsc.edu/
Human	Cpt1a-intron	CTGCTGGAGATCCAACCTTC	TTGATTGCTTAACACAAGTAAGGAC	https://genome.ucsc.edu/
Human	Acox1-intron 1	GTGATTCAGGGAGGGTGGAAC	CTGGCTGCGAGTGAGGAAG	(5)

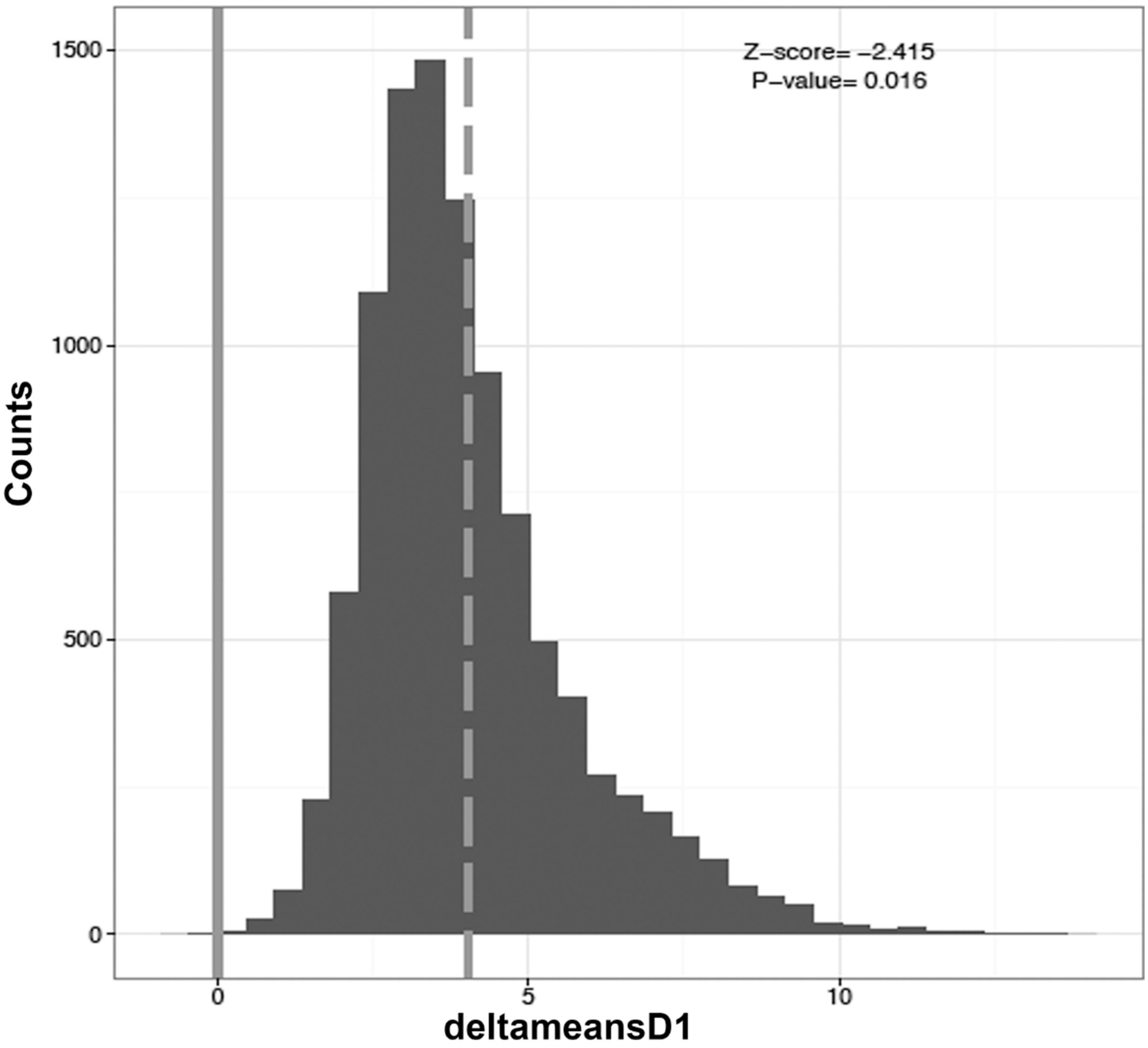

Supplementary Figure S1: Identifying PPARα Targets from IPA and array data. Average fold change of PPARα targets is significantly different from random sample. The difference between the mean fold change of 56 downstream targets of PPARα and 56 randomly selected genes (DeltaMean) was calculated 10,000 times resulting in the distribution shown. The z-score and *p*-value were calculated for the difference between the expected DeltaMean (0, solid line) and the actual DeltaMean (dashed line).

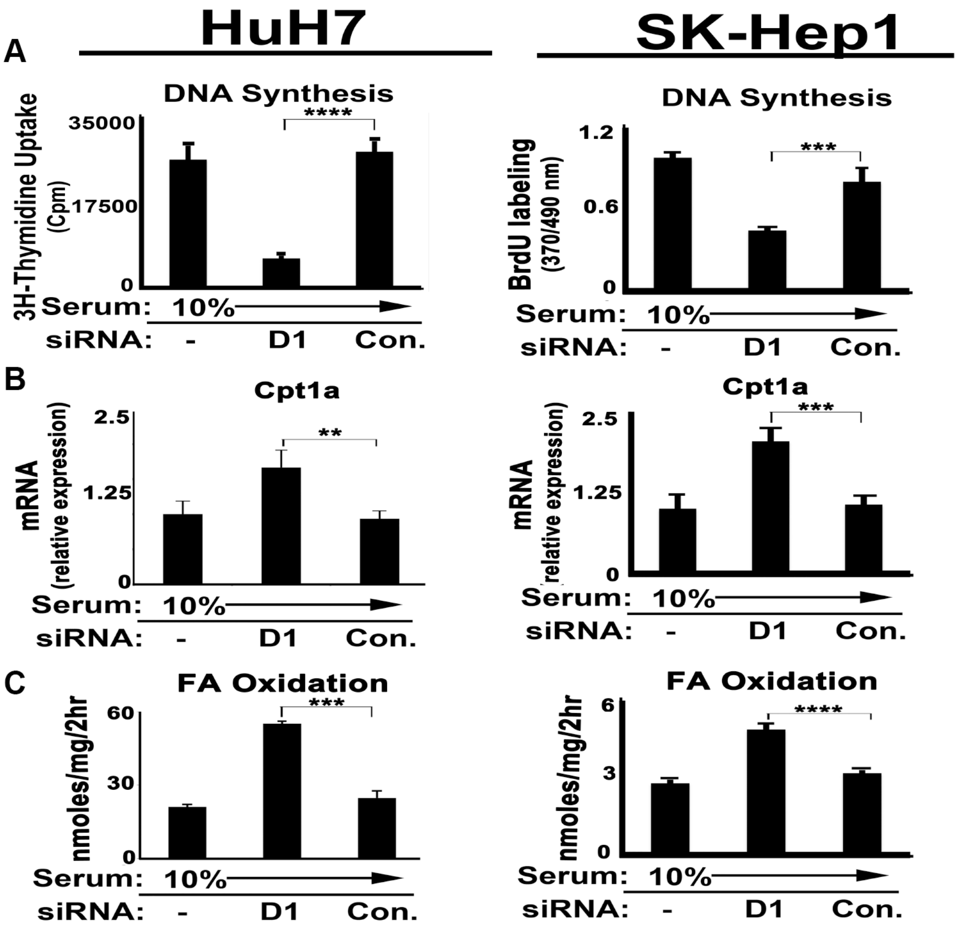

Supplementary Figure S2: Cyclin D1 inhibits PPARα and fatty acid oxidation in other hepatocellular carcinoma cell lines (HuH7 and SK-Hep1). Cells were cultured in the presence of serum (10%) and treated with cyclin D1 (D1) or control siRNA. (**A**) DNA synthesis. (**B**) PPARα-mediated transcript expression. All values were normalized to GAPDH and the relative fold induction was calculated compared to the untreated condition in the presence of serum (10%). (**C**) Fatty acid oxidation.

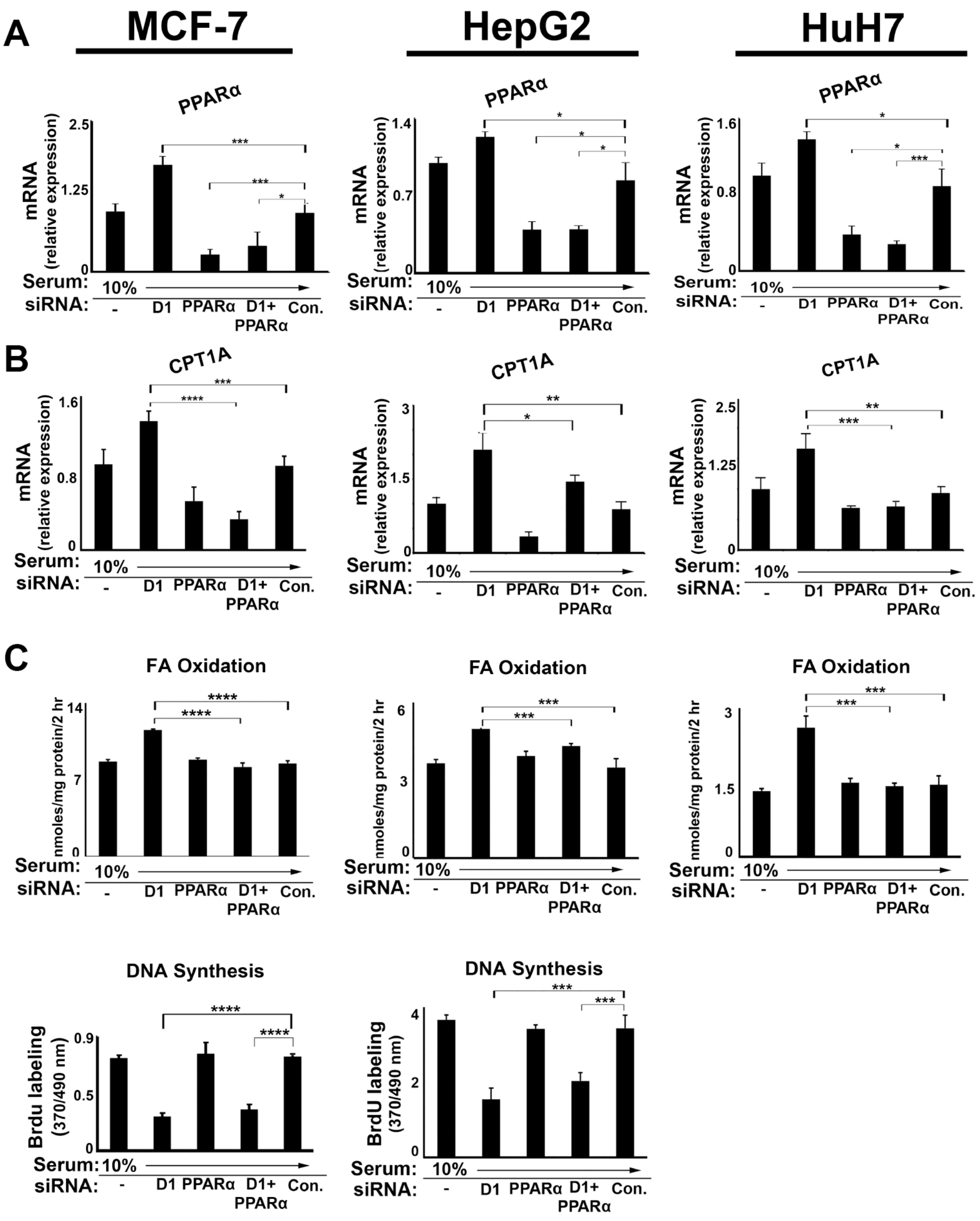

Supplementary Figure S3: Effect of combined cyclin D1 and PPARα knockdown on Fatty acid oxidation and PPARα target genes in malignant cell lines (MCF7, HepG2, and HuH7). Cells were cultured in the presence of 10% serum or FBS and treated with siRNA targeted to cyclin D1, PPARα, or both as indicated. 10 (**A**) RT-PCR for PPARα target gene expression. (**B**) Fatty acid oxidation. (**C**) DNA synthesis.
